# Association of Progressive CD4^+^ T Cell Decline in SIV Infection with the Induction of Autoreactive Antibodies

**DOI:** 10.1371/journal.ppat.1000372

**Published:** 2009-04-10

**Authors:** Takeo Kuwata, Yoshiaki Nishimura, Sonya Whitted, Ilnour Ourmanov, Charles R. Brown, Que Dang, Alicia Buckler-White, Ranjini Iyengar, Jason M. Brenchley, Vanessa M. Hirsch

**Affiliations:** 1 Priority Organization for Innovation and Excellence, Kumamoto University, Kumamoto, Japan; 2 Laboratory of Molecular Microbiology, National Institute of Allergy and Infectious Diseases, National Institutes of Health, Bethesda, Maryland, United States of America; Harvard Medical School, United States of America

## Abstract

The progressive decline of CD4^+^ T cells is a hallmark of disease progression in human immunodeficiency virus (HIV) and simian immunodeficiency virus (SIV) infection. Whereas the acute phase of the infection is dominated by virus-mediated depletion of memory CD4^+^ T cells, chronic infection is often associated with a progressive decline of total CD4^+^ T cells, including the naïve subset. The mechanism of this second phase of CD4^+^ T cell loss is unclear and may include immune activation–induced cell death, immune-mediated destruction, and regenerative or homeostatic failure. We studied patterns of CD4^+^ T cell subset depletion in blood and tissues in a group of 20 rhesus macaques inoculated with derivatives of the pathogenic SIVsmE543-3 or SIVmac239. Phenotypic analysis of CD4^+^ T cells demonstrated two patterns of CD4^+^ T cell depletion, primarily affecting either naïve or memory CD4^+^ T cells. Progressive decline of total CD4^+^ T cells was observed only in macaques with naïve CD4^+^ T cell depletion (ND), though the depletion of memory CD4^+^ T cells was profound in macaques with memory CD4^+^ T cell depletion (MD). ND macaques exhibited lower viral load and higher SIV-specific antibody responses and greater B cell activation than MD macaques. Depletion of naïve CD4^+^ T cells was associated with plasma antibodies autoreactive with CD4^+^ T cells, increasing numbers of IgG-coated CD4^+^ T cells, and increased incidence of autoreactive antibodies to platelets (GPIIIa), dsDNA, and phospholipid (aPL). Consistent with a biological role of these antibodies, these latter antibodies were accompanied by clinical features associated with autoimmune disorders, thrombocytopenia, and catastrophic thrombotic events. More importantly for AIDS pathogenesis, the level of autoreactive antibodies significantly correlated with the extent of naïve CD4^+^ T cell depletion. These results suggest an important role of autoreactive antibodies in the CD4^+^ T cell decline observed during progression to AIDS.

## Author Summary

Despite intensive study, the mechanisms of CD4^+^ T cell depletion in human immunodeficiency virus (HIV) infection remain elusive. The identification of the CD4 receptor as the primary receptor for HIV seemed initially to explain the loss of CD4^+^ T cell lymphocytes in AIDS. However, the number of infected cells at any point in time is insufficient to explain the extent of loss and the slow disease course. While virus-induced cell death might explain early loss of CD4^+^ T cells, such mechanisms were unlikely to explain the slow depletion over the long period leading to AIDS. Thus, mechanisms related to the intense immune activation, also a hallmark of HIV infection, were proposed. Here, we studied a surrogate model for AIDS, simian immunodeficiency (SIV) infection of rhesus macaques, to explore the mechanisms of this second phase of depletion. We found that animals that exhibited slow, progressive loss of CD4^+^ T cells had circulating antibodies that reacted with CD4^+^ T cells and that the levels of these antibodies correlated with the extent of CD4^+^ T cell depletion. These results suggest that autoimmune destruction of CD4^+^ T cells represents a valid mechanism to explore in the pathogenesis of CD4^+^ T cell loss in AIDS.

## Introduction

Progressive CD4^+^ T cell decline in human immunodeficiency virus (HIV) infection, the slow but persistent loss of both naïve and memory CD4^+^ T cells [1]–[3], is an important marker of progression to AIDS. The mechanism of chronic CD4^+^ T cell depletion is not clear since the number of infected cells at any one point in time would not seem to account for the extent of CD4^+^ T cell loss. Such progressive decline in CD4^+^ T cells is also observed in some but not all simian immunodeficiency virus (SIV) macaque models for AIDS [4]–[6]. Recent studies have shown that selective memory CD4^+^ T cell depletion is characteristically observed during the acute stage of SIV- and HIV-infection, presumably through direct killing of CCR5^+^CD4^+^ target cells [7]–[12]. However, early mucosal depletion of memory CD4^+^ T cells is also observed in nonpathogenic infection of natural African monkeys such as African green monkeys and sooty mangabeys [13],[14].

Additional events are therefore required for AIDS pathogenesis in the pathogenic SIV/macaque model and in HIV-infected humans. Many host-mediated mechanisms have been suggested for the depletion of CD4^+^ T cells, including failure of regeneration and homeostasis, immune activation-induced cell death, autoimmune destruction and disruption of the lymphoid microarchitecture by collagen deposition [15]–[18]. Although HIV-1 is named for the immunodeficiency it induces, chronic immune activation is also a characteristic feature of this disease [1], [6], [19]–[22]. The chronic stages of HIV-infection, as well as pathogenic SIV infection, are characterized by generalized lymphadenopathy, immune activation of T-lymphocytes, hyper-gammaglobulinemia, and polyclonal B cell hyperactivity. SIV infection of natural host species, such as sooty mangabeys and African green monkeys, is not associated with the abnormal chronic immune activation and the accelerated T cell turnover seen in pathogenic models, suggesting a critical role of immune activation in the pathogenesis of AIDS [23],[24].

Chronic SIV-infection of macaques provides a relevant and useful model to explore the mechanisms for progressive CD4^+^ T cell loss in AIDS pathogenesis. Earlier studies in our lab demonstrated that the rate of disease progression was associated with distinct patterns of CD4^+^ T cell decline in SIV-infected macaques [6]. SIV-infected macaques that progress rapidly without adaptive immune responses show the most severe memory CD4^+^ T cell loss with relative preservation of naïve cells [6], [25]–[30]. In contrast, a progressive loss of total CD4^+^ T cells including naïve cells is observed during chronic SIV infection of conventional progressors [4]–[6], as is also observed in HIV-1 patients [1]–[3]. Since the level of CD4^+^ T cells in the blood of HIV-infected patients is predictive of the onset of AIDS, we wished to explore this second pattern in greater detail. In this study, we used a SIV/macaque model to explore the mechanisms of CD4^+^ T cell depletion, and found that the progressive loss of CD4^+^ T cells was associated with antibodies that reacted with CD4^+^ T cells. This pattern of depletion was observed in a subset of animals with intense immune activation and autoimmune manifestations consistent with a functional role of these antibodies in the observed CD4 depletion.

## Results

### Two Patterns of CD4^+^ T Cell Depletion in SIV Infection of Rhesus Macaques

**Figure 1:**
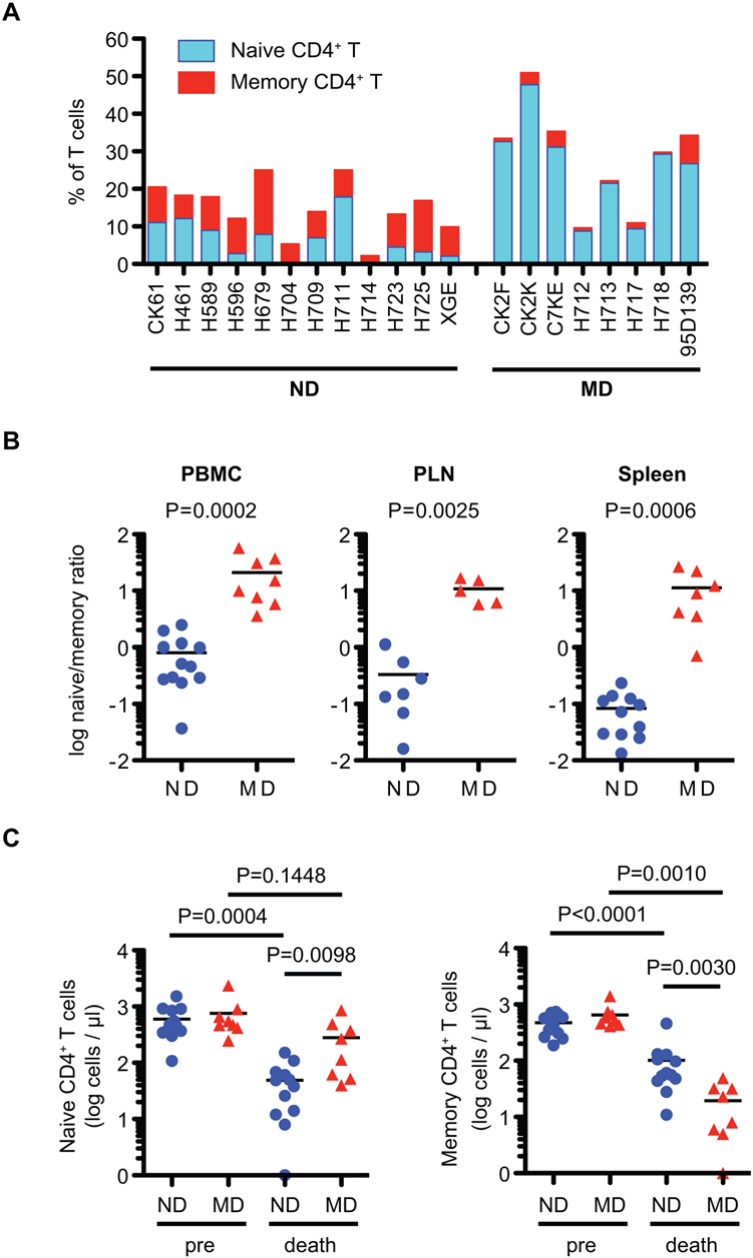
Two patterns of CD4^+^ T cell decline in SIV infection. (A) Classification of the 20 study macaques as memory depletion (MD) or naïve depletion (ND). Naive (blue) and memory (red) CD4^+^ T cells in total T cells in PBMC from 20 macaques at death are shown. Naive (CD95^low^CD28^high^) and memory (CD95^high^CD28^high^ and CD95^high^CD28^low^) subsets of CD4^+^ T cells were analyzed by flow cytometry. (B) Comparison of naive/memory ratio in CD4^+^ T cells in PBMC, PLN, and spleen at death. The ratio was compared between ND (n = 12) and MD (n = 8) in PBMC, ND (n = 7) and MD (n = 5) in PLN, and ND (n = 11) and MD (n = 7) in spleen. (C) Naïve (left) and memory (right) CD4^+^ T cell counts in blood pre-inoculation (pre) and at death. The absolute cell numbers of ND (n = 12) and MD (n = 8) macaques at death were compared by nonparametric Mann-Whitney U test. The cell numbers pre- and at death were compared by Wilcoxon matched pairs t-test.

**Figure 2:**
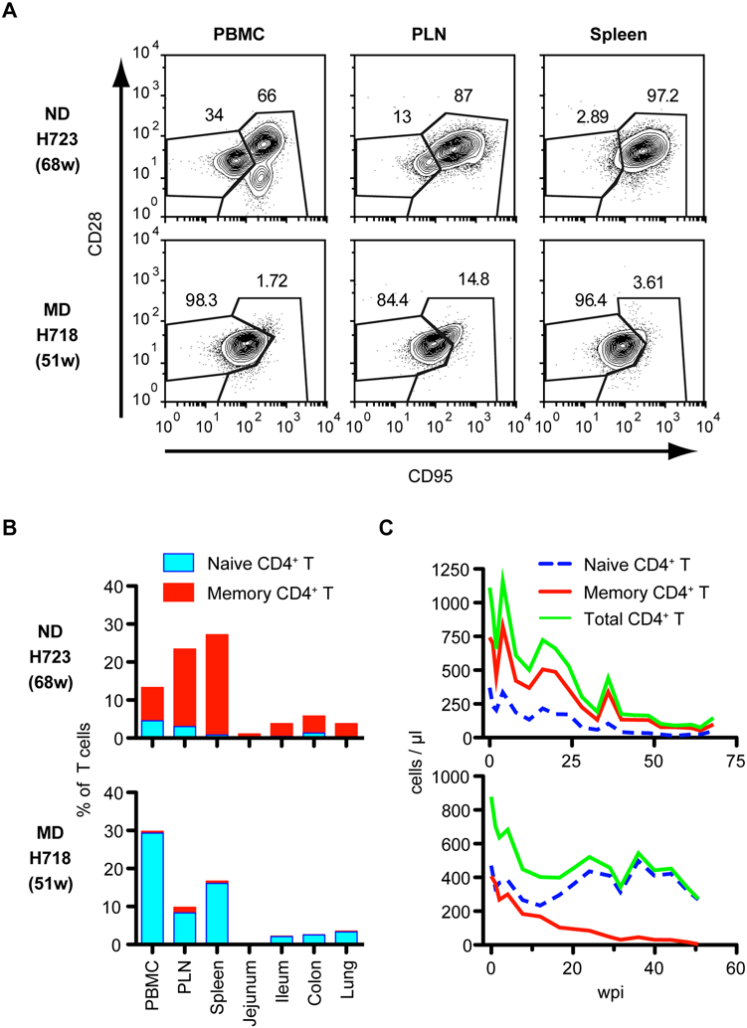
Representative flow cytometry and kinetics of CD4^+^ T cell loss in ND and MD macaques. (A) CD4^+^ T cell subsets of PBMC, PLN, and spleen of macaques H723 and H718 are shown as representatives of ND and MD macaques, respectively. Naive (CD95^low^CD28^high^) and memory (CD95^high^CD28^high^ and CD95^high^CD28^low^) subsets of CD4^+^ T cells were analyzed by flow cytometry. (B) CD4^+^ T cell percentages and their subsets in various tissues from macaques, H723 (upper) and H718 (lower). Naive (blue) and memory (red) CD4^+^ T cells in total T cells are shown. (C) Kinetics of CD4^+^ T cell depletion in H723 (upper) and H718 (lower). Naive (blue dotted line), memory (red), and total (green) CD4^+^ T cell counts in blood are shown.

**Figure 3:**
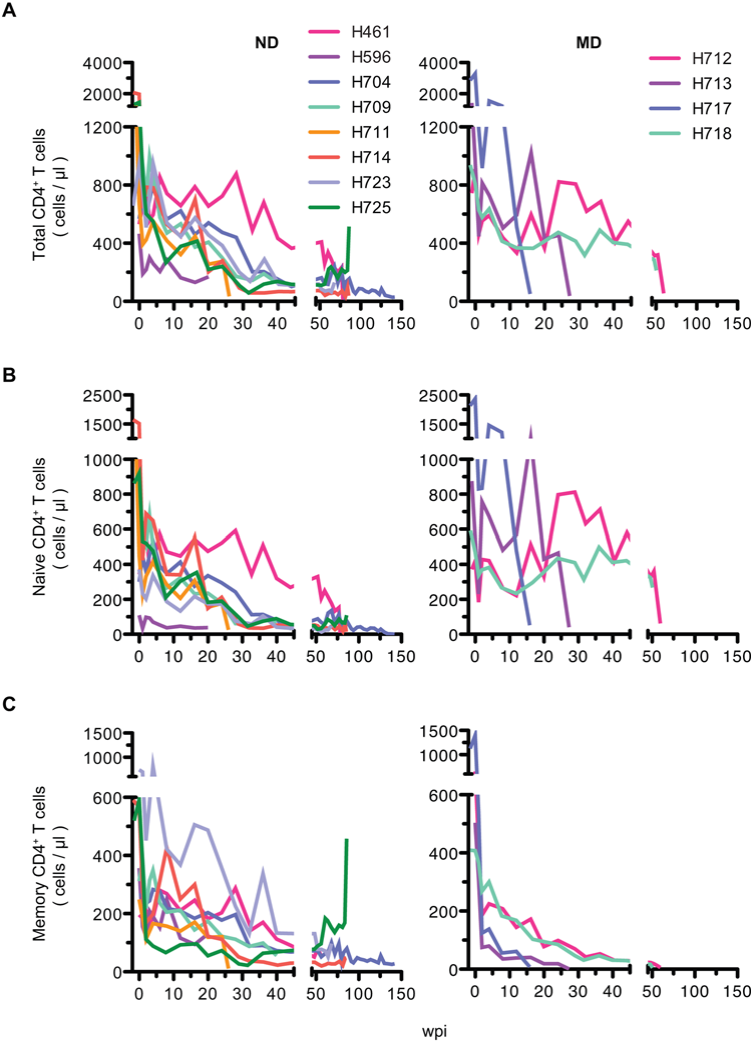
Comparison of the kinetics of CD4^+^ T cell subsets between ND and MD macaques. The kinetics of total (A), naïve (B), and memory (C) CD4^+^ T cell decline in ND (n = 8; left panels) and MD macaques (n = 4; right panels). ND macaques show a progressive and relatively slow decline in all CD4^+^ T cell subsets in the blood during the course of infection, although proportionally more naïve CD4^+^ T cells are lost. In contrast, loss of memory CD4^+^ T cell is a prominent feature observed in MD macaques. A precipitous decline in white blood cell counts terminally in MD macaques results in variable degrees of terminal depletion of total and naïve CD4^+^ T cells.

To clarify the mechanism of progressive CD4^+^ T cell decline in the SIV/ rhesus macaque model, we analyzed CD4^+^ T cell naïve and memory subsets in retrospective samples of blood and various tissues of twenty macaques infected with derivatives of the pathogenic SIVsmE543-3 or SIVmac239 (Table 1). We found that the pattern of depletion of naïve and memory CD4^+^ T cell subsets in terminal blood samples differed substantially, showing two patterns of depletion, predominantly affecting either naïve or memory CD4^+^ T cells (Figure 1A and Figure S1). Animals were classified at death as either ND (naïve depleted) or MD (memory depleted) based upon the ratio of naïve to memory CD4^+^ T cells in the blood (Figure 1B) using a ratio of less than three to identify ND macaques. This classification was confirmed by the naïve to memory ratio in the peripheral lymph nodes (PLN) and spleen of 12 animals (Figure 1B). Twelve macaques showed primarily naïve cell depletion and eight macaques exhibited memory cell depletion (Table 1 and Figure 1A). The naïve to memory ratio of these two groups of animals differed significantly from one another in peripheral blood mononuclear cells (PBMC), PLN and spleen at death (Figure 1B: P = 0.0002, 0.0025 and 0.0006), showing much higher ratios in MD macaques. Comparison of CD4^+^ T cell subsets at pre-inoculation and at death revealed a significant decline in the memory subset in MD macaques and in both subsets in ND macaques (Figure 1C). Figure 2A shows flow cytometric data for a representative macaque of each group. Animal H723 with naïve cell depletion showed a fairly normal representation of subsets in PBMC but preferential depletion of naïve CD4^+^ T cells in lymphoid tissues, where naïve cells are normally abundant in healthy macaques (Figure 2A and 2B). Conversely, H718, a macaque with primarily memory CD4^+^ T cell depletion showed selective depletion of the CD4^+^ memory subset in all tissues (Figure 2A and 2B). As shown in Figure 2C, both memory and naïve CD4^+^ T cell populations gradually declined in H723 during the course of infection, but only the memory population declined in H718. Due to the predominance of naïve cells in peripheral blood in H718, a progressive decline of total CD4^+^ T cells in the chronic phase was only observed in H723. Similar patterns in the kinetics of naïve and memory CD4^+^ T cell loss were observed in the remaining animals of each group. These data were confirmed by examining the kinetics of CD4^+^ T cell declines in 12 individual animals (n = 8 ND and n = 4 MD) as shown in Figure 3. A progressive loss of CD4^+^ T cells, a slow decline of both naïve and memory subsets, was observed in the ND macaques (Figure 3; left panels). Although a precipitous decline in all CD4 subsets (as well as CD8^+^ T cells and B cells, data not shown) occurred terminally in many of the MD macaques possibly due to acute regenerative failure, MD macaques primarily exhibited selective memory CD4^+^ T cell depletion (Figure 3; right panels). Both groups contained macaques infected with a variety of inocula with the exception of SIVsmH635FC-infected macaques that were all classified to the ND group (Table 1). Thus, it appears that the inoculum was not a major factor in determining the type of disease course.

**Table 1:** Clinical and Pathological Outcome of SIV-Infected Macaques

Group	Macaque	SIV Inoculum^a^	Survival (wpi)	Major Pathologic Findings
**ND**	H596	E543-3	23	**Thrombus** (pulmonary artery), LDN
	H711	E543-3+H635FC	26	Mesenteric root torsion, SIVE, GN, LDN
	H709	H635FC	41	**Thrombus** (L. ventricle), enteritis, LDN, SIVE
	CK61	E543-3	56	SIVE, enteritis, cholangitis, LDN
	H589	mac239	60	Enteritis with IA, LDN
	H723	H635FC	68	**Thrombus**, bacterial endocarditis, LDN
	H679	E543-3	73	Renal abscesses, peritonitis, LPD, LDN
	XGE	E543-3	77	**Thrombus** (pulmonary), bacterial pneumonia, IA, LDN
	H461	E543-3	78	Bacterial endocarditis with sepsis, LDN
	H714	H635FC	85	**Thrombus** (vena cava), LDN
	H725	Plasma	86	Disseminated lymphoma, protozoal enteritis, LDN
	H704	H635FC	142	**Thrombus** (Pulmonary artery), LDN
**MD**	CK2K	E543-3	15	SIVE, GN, enteritis
	H717	Plasma	16	SIVE, SIVP, enteritis
	H713	E543-3+H635FC	27	SIVE, protozoal enteritis
	95D139	mac239	28	Enteritis, nephritis (CMV), SIVE
	CK2F	E543-3	31	SIVE, SIVP, GN, enteritis
	CK7E	E543-3	39	SIVE, SIVP, GN, enteritis
	H718	Plasma	51	SIVE, SIVP, Pneumocystis pneumonia
	H712	E543-3+H635FC	60	SIVP, SIVE, enteritis, LDN

a Macaques were inoculated with one of five different inocula, SIVsmE543-3 (E543-3), SIVsmH635FC (H635FC), SIVmac239 (mac239), or plasma from SIVsmH445-infected macaques, H635+H631 (plasma).

LDN, lymphadenopathy; GN, glomerulonephritis; SIVE, SIV encephalitis; SIVP, SIV pneumonia; IA, intestinal amyloidosis; LPD, lymphoproliferative disorder.

**Figure 4:**
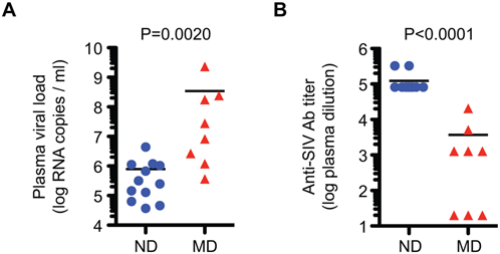
Significant differences in plasma viral load and SIV-specific antibody responses in ND and MD macaques. (A) Plasma viral loads at death were compared between ND (n = 12) and MD (n = 8) macaques. (B) Anti-SIV antibody titers at death were compared between ND (n = 12) and MD (n = 8) macaques by ELISA. Comparisons were performed using the nonparametric Mann-Whitney U test.

These two groups of macaques differed significantly in terms of viral replication and humoral immune responses. As shown in Figure 4A, plasma viral load at the time of death was significantly higher in MD than ND macaques. Conversely, anti-SIV specific antibody titers, as measured by ELISA, were significantly higher in ND than MD macaques (Figure 4B). The higher viral load, low antibody responses and profound memory CD4^+^ T cell depletion seen in the MD macaques, are all characteristic of a syndrome of rapid progression (RP) macaques that is commonly observed in a subset of macaques infected with pathogenic strains of SIV [25]–[30]. Consistent with this similarity, MD macaques included five RP macaques and three macaques with a longer disease course (Table 1). In contrast, none of the ND macaques were typical rapid progressors as defined by lack of SIV-specific antibody responses. However, in at least seven of the ND animals, the disease course was interrupted prior to the development of opportunistic infections by adverse events such as thrombosis. SIV-specific cellular immune responses were not assessed in this cohort but are likely to provide another potential discriminator between these two groups since rapid progression is also associated with failure to maintain SIV-specific CTL responses [30]. The median survival of the MD group was significantly shorter than the ND group (29.5 and 70.5 weeks respectively, P = 0.0135). In terms of pathologic outcome, opportunistic infections were observed in both ND and MD macaques. As expected from the association of SIV encephalitis and pneumonia with rapid progression, these findings were more common in the MD group [25]. A pathologic finding that was unique to the ND group was the occurrence of thrombosis of major vessels such as the pulmonary artery, and vena cava; these events resulted in the acute death of six of the animals (H596, H704, H709, H714, H723 and XGE) in this group.

### ND Macaques Show Generalized Immune Activation

**Figure 5:**
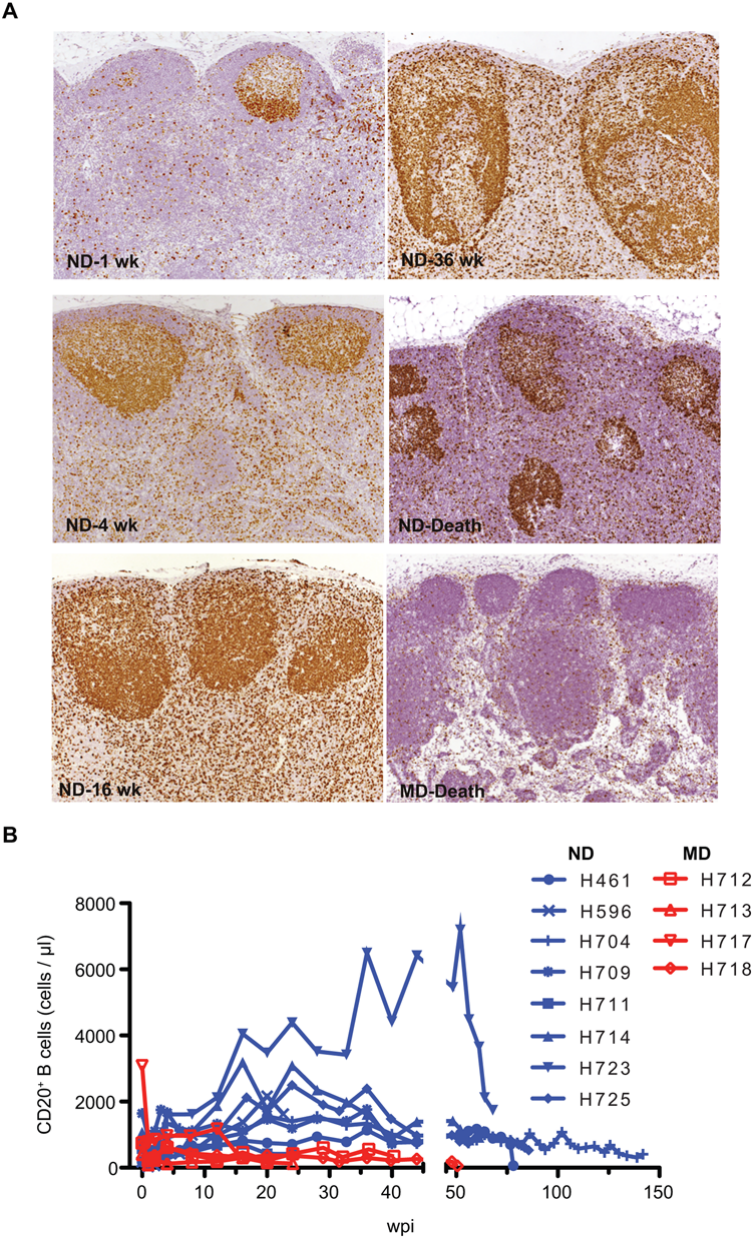
Increased lymphocyte proliferation in lymph nodes and B cell lymphocytosis in ND macaques. (A) Proliferation of lymphocytes analyzed by immunohistochemistry for Ki-67 expression in lymph node biopsies collected at 1, 4, 16, 36 weeks post-inoculation and death of a representative ND macaque (H709) and death of a representative MD macaque (H717). Ki-67 staining of proliferating B cells in germinal centers became increasingly more intense accompanied by an increase in the number Ki-67^+^ cells in the paracortex (T cell area). Germinal centers became larger with time in the ND macaque. This contrasted with the minimal proliferation observed in the lymph nodes of the representative MD macaque. (B) Analysis of CD20^+^ B cells in the blood in ND macaques (n = 8) and MD macaques (n = 4) showed B cell lymphocytosis in many of the ND macaques.

**Figure 6:**
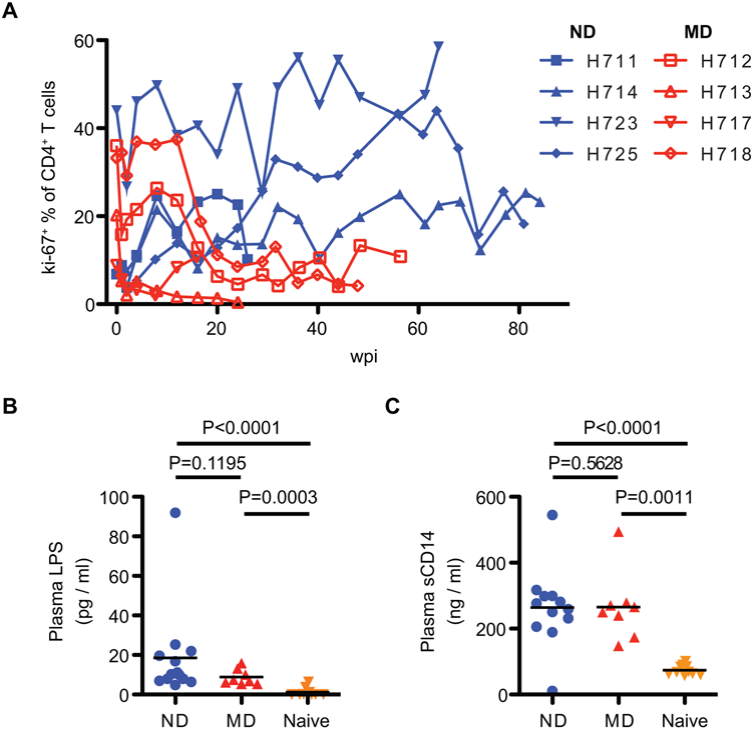
Proliferation of CD4^+^ T cells and microbial translocation in SIV-infected macaques. (A) Proliferation of CD4^+^ T cells was analyzed by Ki-67 coexpression. Kinetics of percentage of Ki-67^+^CD4^+^ T cells in ND (blue) and MD (red) macaques are shown with increasing percentages in ND macaques over the course of infection. (B) Plasma LPS levels were determined in ND (n = 12), MD (n = 8), and SIV-naïve (n = 10) macaques. LPS levels in ND and MD macaques at death were significantly higher than naïve macaques. (C) Plasma levels of sCD14 in ND and MD macaques. LPS and sCD14 levels were compared by nonparametric Mann-Whitney U test.

Secondary indicators of immune activation such as intestinal amyloidosis (n = 2), generalized lymphadenopathy (n = 12) and lymphoproliferative syndrome (n = 1), were commonly observed in the ND group (Table 1). As a measure of lymphocyte proliferation in lymph nodes, immunohistochemistry for Ki-67 expression was performed on sequential lymph node biopsies. As shown in Figure 5A, this analysis demonstrated increasing proliferation in T cell areas and the germinal centers of ND macaques. The development of follicular hyperplasia was a particularly prominent feature of lymphoid tissue in ND macaques and this was often accompanied by increasing numbers of B cells in the blood (Figure 5B). Although immune activation, as indicated by increased levels of Ki-67^+^ CD4^+^ T cells, was observed early in infection in all of the animals, the number of proliferating CD4^+^ T cells, were higher in ND than MD macaques during the chronic phase of infection (Figure 6A). To examine whether the immune activation in ND macaques was induced by increased microbial translocation across the intestinal lumen [19], we measured lipopolysaccharide (LPS) levels in terminal plasma samples (Figure 6B). Both the ND and MD animals showed elevated levels of LPS (mean; 18.6 and 8.8 pg/ml, respectively) as compared to uninfected rhesus macaque plasma LPS (mean; 1.14 pg/ml). An outlier with extremely elevated LPS resulted from gram-negative bacterial sepsis (H679). While there was no statistical significant difference, LPS levels in ND macaques did trend higher than in MD macaques. The level of soluble CD14, which is secreted from CD14^+^ monocyte/macrophages in response to chronic LPS stimulation, was also elevated in both groups (Figure 6C), consistent with significant microbial translocation in both groups. The lack of proliferation of CD4^+^ T cells in MD macaques terminally suggests that these animals have lost the ability to respond to LPS stimulation due to profound immunosuppression.

### Autoreactive Antibodies and Autoimmune Sequelae in ND Macaques

**Figure 7:**
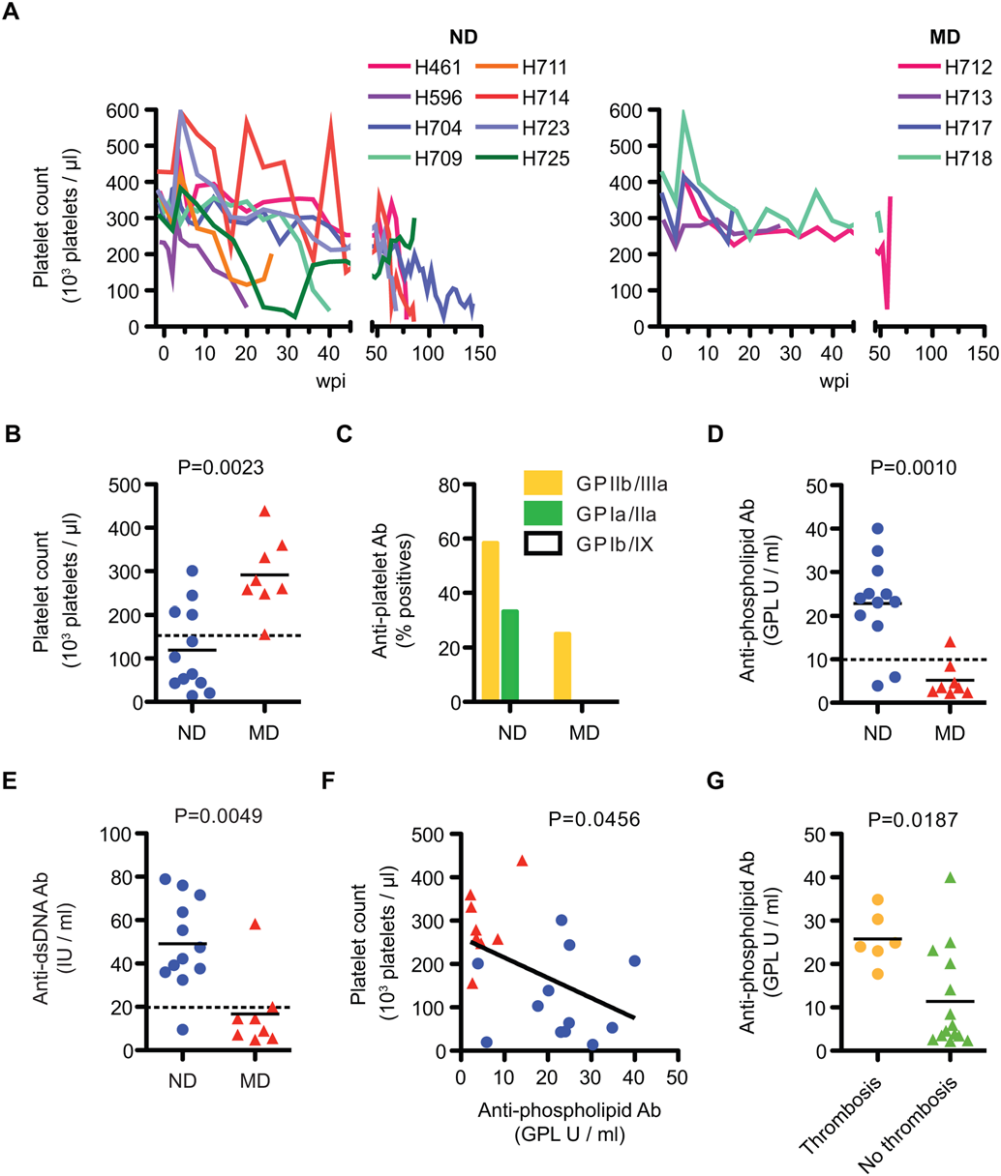
Autoimmune manifestation and autoantibodies in SIV-Infected macaques. (A) Progressive decline of platelets in the blood in ND macaques (n = 8, left), contrasted with more stable levels in MD macaques (n = 4, right). (B) Platelet counts in blood at death were significantly different between ND (n = 12) and MD (n = 8) macaques. (C) A trend to higher frequency of anti-platelet antibodies was observed in plasma samples at death in ND (n = 12) as compared to MD (n = 8) macaques. (D) Anti-phospholipid (aPL) Ab titers at death were significantly higher in ND (n = 12) than in MD (n = 8) macaques. (E) Higher levels of anti-dsDNA Ab titer at death in ND (n = 12) versus MD (n = 8) macaques. (F) A significant correlation was observed between aPL titer and platelet count. Platelet count and aPL titer at death were plotted using ND (blue circles, n = 12) and MD (red triangles, n = 8) macaques. A linear regression analysis indicated a regression line with a slope = −4.66±2.17 (R^2^ = 0.204). (G) Anti-phospholipid antibody titers at death were compared between macaques with thrombosis (n = 6) and without thrombosis (n = 14). Macaques with thrombosis showed a significantly higher level of aPL antibodies.

As is also commonly observed in HIV-infection [31],[32], many SIV-infected macaques exhibited thrombocytopenia during their disease course [33],[34]. As shown in Figure 7A, a progressive decline was observed throughout the course of infection in ND macaques and the platelet count at the time of death was significantly lower in ND than MD macaques (Figure 7B). Antibodies against platelet glycoproteins were detected in ND macaques more frequently than in MD macaques (Figure 7C). Similar to immune thrombocytopenia in HIV infection, anti-GPIIIa was the most frequently observed platelet autoantibody [31]. Many of the macaques with severe thrombocytopenia died suddenly due to thrombosis of a major vessel and this finding was unique to ND macaques. Although such thrombotic events and associated arteriopathy are commonly associated with chronic SIV-infection, their etiology is unclear. In HIV-infected patients, a hypercoagulable state induced by autoantibodies to phospholipid (aPL) and other clotting factors can lead to thrombotic events [35]. We therefore examined plasma samples from these macaques for the presence of aPL antibodies. As shown in Figure 7D, a significant increase in aPL was observed in ND but not MD macaques. ND macaques also exhibited elevated dsDNA antibodies, suggesting a generalized autoimmune dysregulation in ND macaques (Figure 7E). Consistent with a functional consequence in macaques, aPL titers inversely correlated with platelet counts and aPL titers were significantly higher in macaques with catastrophic thrombotic events (Figure 7F and 7G).

### Association of Progressive CD4^+^ T Cell Loss with Autoreactive Antibodies

**Figure 8:**
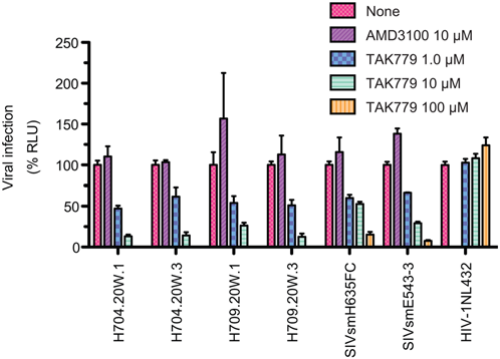
Viruses from ND macaques use CCR5 as co-receptor. Co-receptor usage of viruses in ND macaques and parental SIV strains was analyzed using CCR5 and CXCR4 antagonists, TAK779 and AMD3100, respectively. TZM-bl cells were incubated with pseudotyped viruses in the absence or presence of the designated concentration of antagonist. The mean percentage of the control luciferase activity is shown with standard errors. Pseudotyped viruses were prepared using Env of SIVsmH635FC and SIVsmE543-2, and two Env clones each from H704 and H709. The infection with pseudotyped viruses with Env from H704 and H709 were inhibited by TAK779, but not AMD3100, similar to parental SIVsmH635FC and SIVsmE543-3. In contrast, the X4-tropic HIV-1NL43-3 was inhibited by AMD3100, but not TAK779.

The memory CD4^+^ T cell loss in MD macaques would appear to be mainly explained by direct cell killing of CCR5^+^CD4^+^ target cells that is compounded by insufficient production [7],[8],[29]. In contrast, the loss of naïve CD4^+^ T cells in ND macaques was not readily explained by virus killing since these cells primarily express CXCR4, not CCR5 [11] and SIV strains generally use CCR5 as their major co-receptor. The emergence of a CXCR4-tropic SIV has only been observed once in macaques and was associated with extensive substitutions in the V3 region of Env [29],[36]. Similarly, emergence of X4-tropic SIVs in sooty mangabeys was associated with changes within the V3 loop [37]. Our prior sequence analysis of sequential plasma virus from a number of these animals did not show evolution of this region of Env to X4 tropism [27] (Figure S2). Therefore we used envelope clones from 20 wpi plasma of two of these animals (H704 and H709) to evaluate whether emergence of a X4 variant explained the naïve CD4^+^ T cell depletion observed in these animals by this time point. Virus pseudotypes produced by cotransfection of appropriate envelope clones with pSG3ΔEnv, a Rev expression plasmid, were assayed for sensitivity to the CCR5 and CXCR4 antagonists, TAK779 and AMD3100 in TZM-bl cells. As observed in Figure 8, the parental viruses SIVsmH635 and SIVsmE543-3 and clones derived from two ND macaques were inhibited only by the CCR5 antagonist, consistent with maintenance of CCR5 as their major co-receptor. This contrasted with the expected inhibition of the CXCR4-tropic HIV-NL4-3 by AMD3100. To validate this finding, we also evaluated the infection frequency by quantitative real time PCR for SIV DNA within sorted populations of naïve and memory CD4^+^ T cells from samples of spleen collected at necropsy of five of the ND macaques. By this time point, only one sample (H709) had sufficient naïve cells (8.8%) for accurate cell sorting and qPCR. For this sample, the ratio of SIV gag copies per cell number was 29% of memory CD4^+^ T cells versus 5% of naïve CD4^+^ T cells, consistent with preferential infection of memory CD4^+^ T cells. Hence the emergence of CXCR4-tropic SIV within ND macaques did not appear to explain the depletion of naïve CD4^+^ T cells in at least two of these animals. Further more detailed analysis would be necessary to totally eliminate a role for X4 viruses.

**Figure 9:**
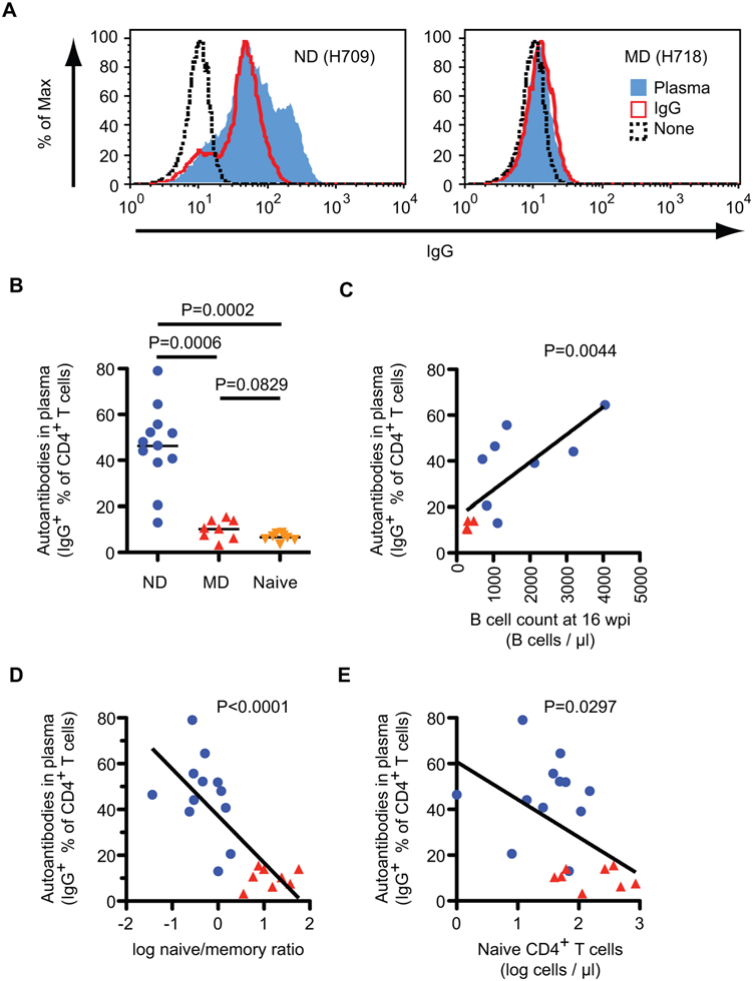
Plasma antibodies autoreactive to CD4^+^ T cells in ND macaques. (A) Antibodies against CD4^+^ T cells were analyzed using plasma and purified IgG from infected macaques. PBMC from SIV-uninfected macaques were incubated with plasma (5-fold–diluted, blue line) or purified IgG (1.5 mg/ml, red line) from ND (H709, left) or MD (H718, right) macaques, and stained with antibodies against monkey IgG (FITC), CD3 (PerCP-Cy5.5), and CD4 (APC). A histogram of monkey IgG is shown after gating CD3^+^CD4^+^ cells. Dashed line; IgG reactivity of untreated PBMC. (B) Autoantibodies in terminal plasma samples were compared among ND (n = 12), MD (n = 8), and SIV-naïve (n = 8) macaques. Percentages of IgG^+^ cells in CD4^+^ T cells were determined by the Overton cumulative histogram subtraction using untreated PBMC as a control. (C) Correlation between autoantibodies to CD4^+^ T cells in plasma and B cell count in blood at 16 wpi. Percentages of IgG^+^ cells in CD4^+^ T cells and B cell counts were plotted using ND (blue circles, n = 8) and MD (red triangles, n = 4) macaques. A linear regression analysis indicated a regression line with a slope = 0.012±0.003 (R^2^ = 0.572). (D) Correlation between autoantibodies to CD4^+^ T cells in plasma and log naïve/memory ratio in CD4^+^ T cells at death. Percentages of IgG^+^ cells in CD4^+^ T cells and log naïve/memory ratio in CD4^+^ T cells were plotted using ND (blue circles, n = 12) and MD (red triangles, n = 8) macaques. A linear regression analysis indicated a regression line with a slope = −20.5±4.04 (R^2^ = 0.588). (E) Correlation between autoantibodies to CD4^+^ T cells in plasma and log naïve CD4^+^ T cell number at death. Percentages of IgG^+^ cells in CD4^+^ T cells and log absolute naïve CD4^+^ T cell number were plotted using ND (blue circles, n = 12) and MD (red triangles, n = 8) macaques. A linear regression analysis indicated a regression line with a slope = −16.5±6.97 (R^2^ = 0.237).

**Figure 10:**
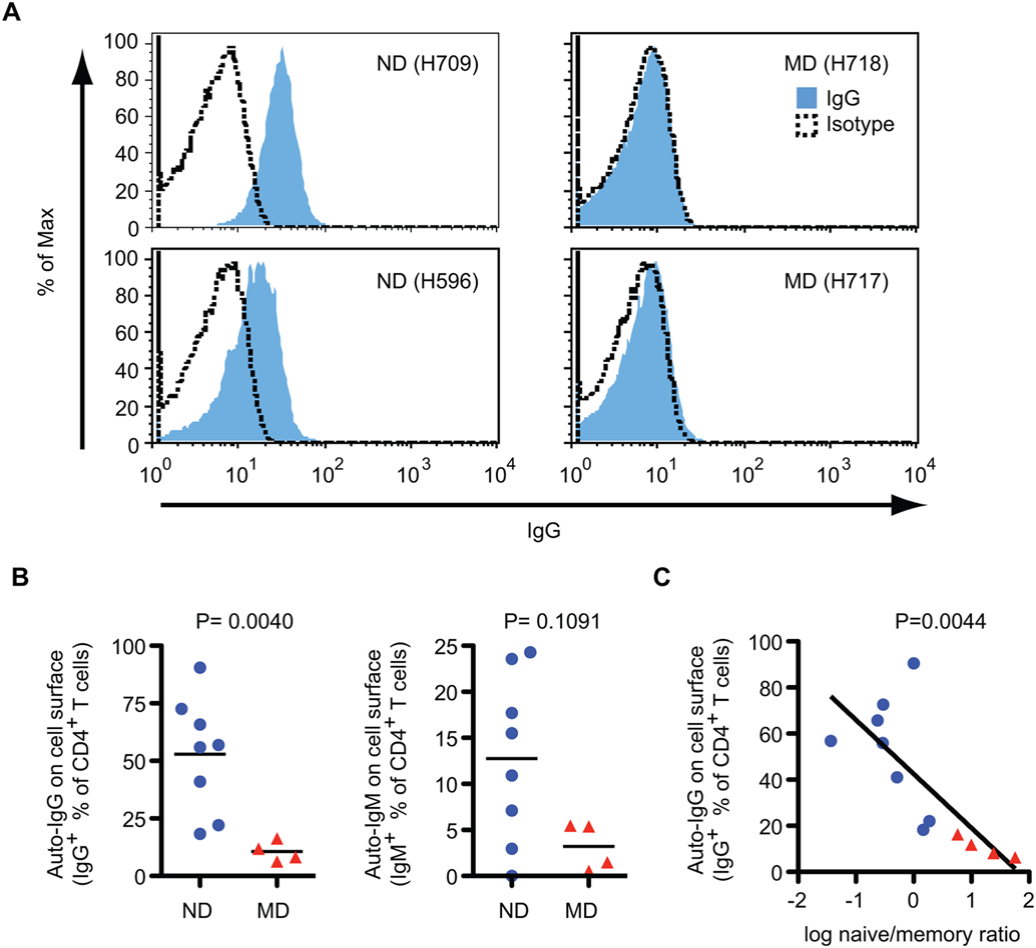
Autontibodies on the surface of CD4^+^ T cells in ND macaques. (A) Autoantibodies on the surface of CD4^+^ T cells from ND (H709, left) and MD (H718, right) macaques in representative flow cytometric analysis. Frozen PBMC samples at death were stained with antibodies against CD4 (FITC), human IgG (PE), 7-AAD (PerCP), and CD3 (APC). Autoantibodies were examined by anti-IgG reactivity (blue) on the surface of live CD4^+^ T cells (7-AAD^−^CD3^+^CD4^+^). Dashed line, staining with IgG_1_ isotype control (PE) instead of anti-human IgG (PE). (B) The percent of autoantibody positive CD4^+^ T cells at death were compared between ND (n = 8) and MD (n = 4) macaques. Percentages of IgG^+^ (left) or IgM^+^ (right) CD4^+^ T cells were determined by the Overton cumulative histogram subtraction using a mouse IgG_1_ isotype control. (C) Correlation between autoantibodies on CD4^+^ T cells and log naïve/memory ratio in CD4^+^ T cells at death. Percentages of IgG^+^ cells in CD4^+^ T cells and log naïve/memory ratio in CD4^+^ T cells were plotted using ND (blue circles, n = 8) and MD (red triangles, n = 4) macaques.

The heightened immune activation and the propensity for autoimmune manifestations in the ND macaques led us to explore the possibility that autoimmune mechanisms might account for the loss of naive CD4^+^ T cells in these animals. We therefore examined terminal plasma samples for antibodies that bound the surface of CD4^+^ T cells from healthy, uninfected donor macaques. In addition, terminal CD4^+^ T cells of study macaques were analyzed for surface IgG and IgM. As shown in Figure 9A and 9B, antibodies that bound CD4^+^ T cells were detected exclusively in plasma or purifed IgG from ND macaques. Moreover, the level of anti-CD4^+^ T cell IgG correlated significantly with B cells count at 16 wpi, when activation of B cells were apparent (Figure 9C and Figure 5B). Most importantly, the level of anti-CD4^+^ T cell IgG correlated significantly with a lower naïve/memory ratio of CD4^+^ T cells, an indicator of naïve depletion (Figure 9D) as well as with absolute naïve CD4^+^ T cell counts at death (Figure 9E). We next examined T cells from PBMC samples from ND and MD macaques for the presence of surface-bound IgG by flow cytometry. In vivo antibody binding was also exclusively detected on the surface of CD4^+^ T cells from ND macaques, as shown by representative flow cytometry plots in Figure 10A. The percentage of CD4^+^ T cells with surface IgG was significantly higher in the ND versus the MD group (Figure 10B). In vivo binding of IgM was also detected on the CD4^+^ T cells from ND macaques, though there was no significant difference between the two groups (Figure 10B). Similar to plasma antibodies, a significant inverse correlation was observed between the percent of IgG^+^CD4^+^ T cells and the memory/naïve CD4^+^ T cell ratio (Figure 10C); macaques with higher anti-CD4^+^ T cell antibodies had lower naïve/memory ratio, indicative of naïve CD4^+^ T cell depletion.

**Figure 11:**
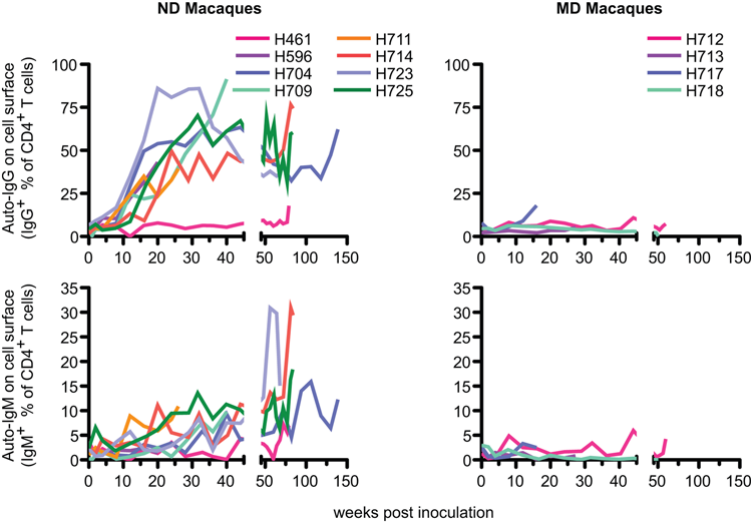
The kinetics of induction of antibodies autoreactive to CD4^+^ T cells. Kinetics of IgG (upper) and IgM (lower) on the surface of CD4^+^ T cells from ND (left, n = 8) and MD (right, n = 4) macaques are shown graphically. Frozen PBMC samples were stained with antibodies against CD4 (FITC), human IgG, IgM, or IgG_1_ isotype control (PE), 7-AAD (PerCP), and CD3 (APC). Percentages of IgG^+^ or IgM^+^ cells in live CD4^+^ T cells (7-AAD^−^CD3^+^CD4^+^) were determined by the Overton cumulative histogram subtraction using mouse IgG_1_ isotype control.

**Figure 12:**
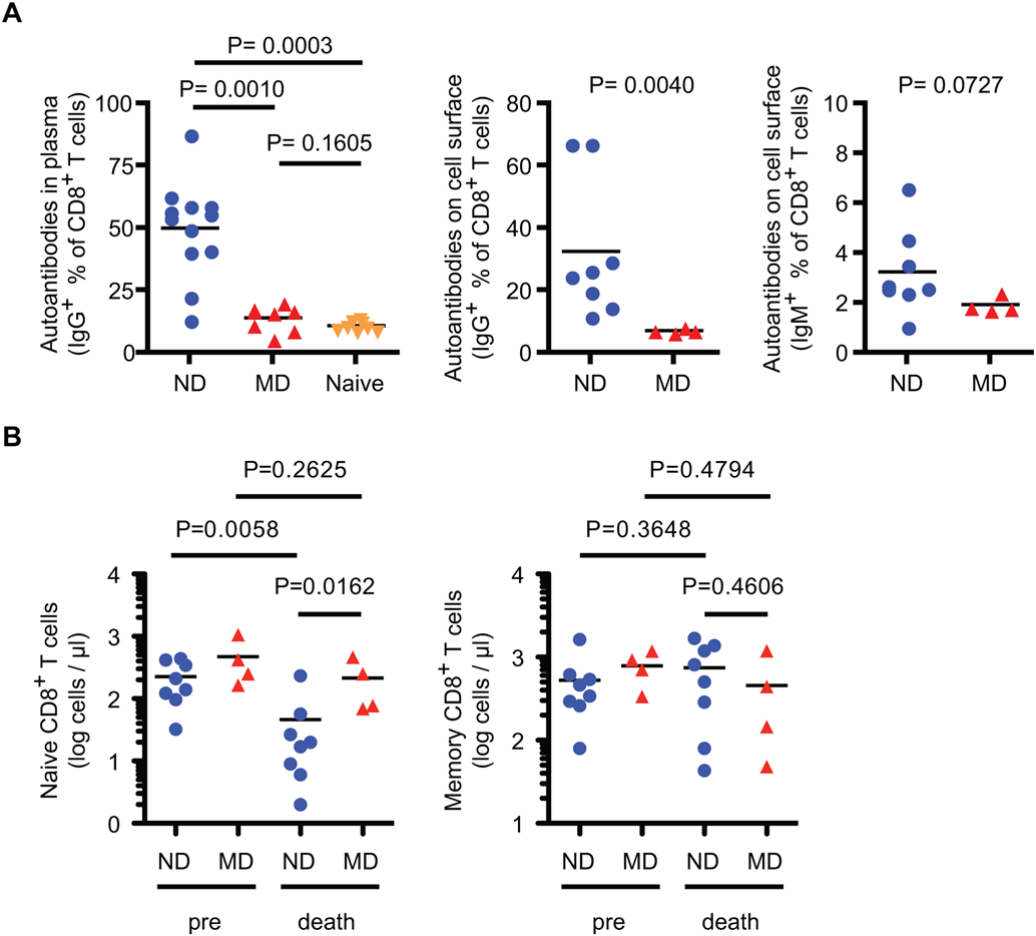
Antibodies autoreactive to CD8^+^ T cells and decline of naïve CD8^+^ T cells in ND macaques. (A) Autoantibodies against CD8^+^ T cells in plasma (left) were analyzed using CD4^−^ T cells, and were compared among ND (n = 12), MD (n = 8), and SIV-uninfected (n = 8) macaques, as described in Figure 9. Autoantibodies on the surface of CD8^+^ T cells (right two panels) were analyzed using CD8^+^ T cells, and were compared between ND (n = 8) and MD (n = 4) macaques, as described in Figure 10. (B) Naive and memory CD8^+^ T cell count in blood at death. The absolute numbers of cells were compared between ND (n = 8) and MD (n = 4) macaques, as described in Figure 1C.

The kinetics of the development of CD4^+^ T cell bound IgG and IgM were analyzed on cryopreserved PBMC samples (Figure 11). IgG was detected on the surface of CD4^+^ T cells in most of the ND macaques by eight or 12 wpi, peaking generally around 30 wpi. The emergence of autoantibodies was coincident with the development of naïve CD4^+^ cell deletion. IgM was also detected on CD4^+^ T cells but at much lower levels, gradually increasing in most of ND macaques. Similar results were obtained from the analyses of CD8^+^ T cells; antibodies that bound CD8^+^ T cells from SIV-uninfected macaques were detected in plasma and on the surface of CD8^+^ T cells of ND macaques (Figure 12A) but the levels were lower than that observed on CD4^+^ T cells. Depletion of naïve CD8^+^ T cells, which is common in HIV-infected patients [1],[2], was specifically observed in ND macaques (Figure 12B), suggesting that the naïve subset depletion is associated with autoreactive antibodies in both T cell populations.

## Discussion

In the present study, autoreactive antibodies to platelet glycoproteins, phospholipids, dsDNA and surface antigens on T cells were observed in a subset of SIV-infected macaques that showed primary depletion of naïve CD4^+^ T cells. These antibodies were associated with pathologic consequences, such as thrombocytopenia, thrombosis and naïve cell depletion of both CD4^+^ and CD8^+^ T cells in blood and tissues. Our data are consistent with a potential role of these antibodies in CD4^+^ T cell depletion in SIV infection and has additional implications for the pathogenesis of AIDS in humans.

The defining feature of HIV-associated disease is the slow loss of CD4^+^ T cells in the chronic phase of infection and resulting immunodeficiency [1]–[3]. However, a heightened state of generalized immune activation of T [1],[19],[20] and B cells [21],[22] is also a prominent feature of HIV-infection. Indeed, the extent of immune activation in HIV-infection is an independent indicator of disease progression, as informative as plasma viral load [20] and CD4^+^ T cell counts. Moreover, the extent of immune activation is a striking difference between pathogenic and nonpathogenic infections with SIV [23],[24]. The exact mechanisms whereby immune activation contributes to AIDS progression have not been clearly defined. Immune activation is presumed to lead to CD4^+^ T cell depletion through indirect mechanisms such as providing new activated CD4^+^ T cell targets and activation-induced apoptosis. Our study provides evidence that the destruction of CD4^+^ T cells by autoreactive antibodies may be a third mechanism that also exerts its action through immune activation. Indeed, the extent of immune activation in HIV/SIV infection has been strongly associated with the development of autoantibodies and various autoimmune diseases [31],[32],[38]. One such study observed a correlation with autoantibodies to nuclear, smooth muscle, and thyroid antigens with a lower CD4^+^ T cell count and increased mortality, consistent with prognostic value [38].

Antibodies to an array of self-antigens and resultant autoimmune disease manifestations are frequently observed in HIV infection [31],[32],[38]. Primary clinical manifestations which have been observed in HIV-infection include thrombocytopenia, anemia, systemic vasculitis, Reiter's syndrome, polyarthritis, Sjogren's syndrome and antiphospholipid syndrome (APS) [32]. Autoantibodies and autoimmune diseases have also been demonstrated in SIV-infected macaques [33],[34],[39]. In the present study, we observed an association of anti-platelet and antiphospholipid antibodies with clinical autoimmune disease, specifically thrombocytopenia and APS in macaques. These results suggest that the production of these antibodies had functional and clinical consequences in these macaques. The mechanisms proposed for the generation of autoreactive antibodies in HIV/SIV infection include the loss of regulatory T cells [40], production of anti-idiotype antibodies [41], and molecular mimicry of gp120 or gp41 with self antigens [42],[43]. All of these implicate immune activation, especially B lymphocyte hyperactivation with hypergammaglobulinemia [21],[22].

The hypothesis that autoimmune mechanisms play a role in AIDS pathogenesis is not a new idea. Anti-CD4^+^ T cell antibodies were reported soon after identification of AIDS, and a role in CD4^+^ T cell decline was suggested [44]–[46]. This avenue of research has not been adequately studied primarily since the field's mechanistic focus shifted to the direct effects of the virus on destruction of CCR5^+^CD4^+^ T cells [7]–[12] and the indirect effects of immune activation [15]–[18]. Additionally, although the presence of these antibodies is not at issue, it has been difficult to clearly show cause and effect. Several studies have shown an association between anti-CD4^+^ T cell antibodies with CD4 T^+^ cell depletion and disease progression [40], [47]–[49], suggesting the potential contribution to AIDS pathogenesis. However, none of these studies have clearly identified the mechanism within, and the subset in CD4^+^ T cells that is depleted. Our study demonstrated an association of anti-CD4^+^ T cell antibodies with loss of naïve CD4^+^ T cells, which is responsible for the progressive CD4^+^ T cell decline during chronic SIV/HIV infection. This suggests that the induction of anti-CD4^+^ T cell antibodies is one of the important factors causing a slow depletion of CD4^+^ T cells over the long asymptomatic period leading to AIDS. This mechanism probably dominates during the earlier and mid stages of HIV infection when the immune system is highly dysregulated but prior to the onset of severe immunodeficiency.

Potential mechanisms by which antibodies could mediate CD4^+^ T cell loss including complement-mediated lysis, phagocytosis or antibody-dependent cell-mediated cytotoxicity [45],[47],[50] will require evaluation in these animals. In addition, autoantibodies may affect the production and differentiation of naïve T cells in thymus and bone marrow [4],[51],[52]. Since autoantibodies were specifcially associated with the loss of naïve CD4^+^ T cells rather than memory cell, this represents a distinct mechanism from the recently reported decline of the central CD4^+^ T cell pool, which is also an important subset for maintaining CD4^+^ T cells [29],[53]. The decline of naïve cells of both CD4^+^ and CD8^+^ T cells in this study and HIV-1-infected patients [1],[2] also suggests that the mechanism for the decline and the self antigens involved may be common to both T cells. Another autoimmune mechanism not evaluated in the present study, was the generation of autoreactive CD8^+^ T cells primed by the release of protein fragments from apoptotic CD4^+^ T cells [54]. MHC alleles may be critical to the induction of autoreactive antibodies, as indicated in a number of autoimmune diseases [55] but were not assessed in this study.

Although further studies are required to define the contribution of autoantibodies to AIDS pathogenesis, our study suggests that such antibodies may act in concert with other mechanisms of CD4^+^ T cell loss, such as immune activation induced cell death and destruction of the lymphoid architecture [15]–[18]. Our data are consistent with a potential role of these antibodies in CD4^+^ T cell depletion in SIV infection and has additional implications for the pathogenesis and treatment of AIDS in humans.

## Methods

### Animals and Viruses

Rhesus macaques (*Macaca mulatta*) of Indian origin were inoculated with molecularly cloned virus stocks generated by transfection of 293T cells, (SIVsmE543-3, SIVsmH635FC, SIVmac239), a combination of SIVsmE543-3 and SIVsmH635FC, or terminal plasma samples from two SIVsmH445-infected macaques, H631 and H635 [6], [26]–[28]. Twenty SIV-infected macaques used in this study consist of the 9 macaques from Kuwata *et al.* [27], 8 macaques from Nishimura *et al.* [6], and 3 macaques infected with plasma from SIVsm-infected macaques. Our criteria for inclusion in the study were lack of prior treatment or vaccination and the availability of terminal blood samples for analysis. EDTA-anti-coagulated blood samples were collected sequentially to examine plasma viral RNA load and lymphocyte subsets. Tissue samples, such as jejunum, ileum, colon, lung, PLN and spleen, were obtained at death from a subset of the animals. Lymphocytes were isolated from the blood, spleen and PLN by Ficoll gradient, and by collagenase treatment from jejunum, ileum, colon and lung [27]. The viral RNA loads in plasma were determined by real-time reverse transcriptase-PCR (RT-PCR) using a Prism 7700 sequence detection system (Applied Biosystems, Foster City, CA), as previously described [27]. All animals were housed in accordance with American Association for Accreditation of Laboratory Animal Care standards. The investigators adhered to the Guide for the Care and Use of Laboratory Animals prepared by the Committee on Care and Use of Laboratory Animals of the Institute of Laboratory Resources, National Resource Council, and the NIAID Animal Care and Use Committee-approved protocols.

### Determination of Co-Receptor Usage of Viruses

Co-receptor usage of viruses infecting ND macaques and parental SIV strains was analyzed using CCR5 and CXCR4 antagonists, TAK779 and AMD3100 [56]–[60]. The Env gene was amplified from plasma samples of H704 and H709 at 20 wpi, and cloned into the expression vector, pcDNA3.1/V5-His TOPO (Invitrogen, Carlsbad, CA), as described [26]. Pseudotyped viruses were prepared by cotransfection of 293T cells with pSG3ΔEnv, Rev expression plasmid and Env expression plasmid using Lipofectamine 2000 (Invitrogen) [61],[62]. After a 1 h incubation of 40 μl of 2.5×10^6^ TZM-bl cells [62]–[64] in the absence or presence of 10 μl of designated concentration of antagonist in 96 well-plate, 45 μl of pseudotyped viruse and 5 μl of 1.875 μg/ml DEAE dextran were added. After over night incubation, 100 μl fresh medium was added to wells. Luminesence was measured after 2 days using Luciferase Assay System (Promega, Madison, WI) and Mithras microplate luminometer (Berthold Technologies, Bad Wildbad, Germany). Infection was performed in triplicate and data expressed as the mean. The following reagents were obtained through the NIH AIDS Research and Reference Reagent Program, Division of AIDS, NIAID, NIH: TAK779, AMD3100, pSG3ΔEnv and TZM-bl cells.

### Flow Cytometric Analysis

Phenotypic analysis of T cells was performed by flow cytometry (FACSCalibur; BD Biosciences, Franklin Lakes, NJ) using FITC, PE, PerCP-Cy5.5 or APC-conjugated monoclonal antibodies against CD3, CD4, CD8, CD28 and CD95 (BD Biosciences), as described [6],[27]. Percentages of naive and memory subsets in CD4^+^ or CD8^+^ T cells were determined using CD28 and CD95. Live cells were gated using 7-AAD in the analyses of viably-frozen cells. Immunoglobulin on the surface of cells was detected by PE-conjugated antibodies against human IgG or human IgM with a mouse IgG_1_ isotype-matched control (BD Biosciences). Since histograms of a control and experimental samples were overlapping, the percentages of IgG^+^ or IgM^+^ cells were determined by the Overton cumulative histogram subtraction, using mouse IgG_1_ isotype control [65]. To detect antibodies to T cells in the plasma, PBMC from SIV-uninfected rhesus macaques (5×10^5^ cells in 50 μl) were incubated for 1 hr at 4°C with 50 μl of plasma (1:5 dilution with PBS) or purified IgG (1.5 mg/ml) from SIV-infected macaques, washed twice with PBS supplemented with 0.1% bovine serum albumin, and stained with antibodies against monkey IgG (FITC), CD3 (PerCp-Cy5.5) and CD4 (APC). FITC-conjugated goat anti-monkey IgG (MP Biomedicals, Solon, OH) was used to detect antibodies in plasma. IgG was purified from plasma samples by Nab Protein G Spin Kit (Thermo Fisher Scientific, Rockford, IL). The percentages of IgG^+^ cells in CD4^+^ T cells were determined by the Overton cumulative histogram subtraction using untreated controls. Data analysis was performed using Flowjo (TreeStar, San Carlos, CA).

### Detection of Antibodies to SIV and Autoantigens

Anti-SIV antibody titers were determined by enzyme immunoassay (EIA), Genetic Systems HIV2 EIA (Bio-Rad Laboratories, Hercules, CA) using serial dilution of plasma samples. Antibodies against three glycoproteins on platelet, GPIIb/IIIa, GPIa/IIa and GPIb/IX, were determined by EIA, PAKAUTO (GTI Diagnostics, Waukesha, WI). Antibodies (IgG) against phopholipid in plasma samples were quantified by anti-phopholipid screen IgG/IgM (ORGENTEC Diagnostika GmbH, Mainz, Germany), which detects antibodies against cardiolipin, phosphatidyl inositol, phosphatidic acid and ß2-glycoprotein I. Antibodies (IgG) against double-stranded DNA were quantified by anti-dsDNA (ORGENTEC Diagnostika GmbH).

### Measurement of Lipopolysaccharide (LPS) and sCD14

Plasma levels of LPS were measured by the Chromogenic end point Limulus amebocyte kit (Cambrex, Charles City, IA) as recommended by the manufacturer. A commercially available ELISA was used to quantify plasma levels of sCD14 (R&D Systems, Minneapolis, MN); each ELISA was performed in duplicate according to the manufacturer's protocol.

### Cell Sorting of Memory and Naïve CD4^+^ T Cells and Quantitative PCR

Cell sorting was accomplished using a FACS Aria cell sorter (BDIS) at 70 lb/in2. FITC, PE, Cy5 PE, and APC were used as the fluorophores. At least 10,000 cells were sorted for PCR and reverse transcriptase PCR analysis. Sorted populations were consistently at least 99.8% pure. Quantification of SIV gag DNA in sorted CD4^+^ T cells was performed by quantitative PCR by means of a 5′ nuclease (TaqMan) assay with an ABI7700 system (Applied Biosystems) as previously described [66]. To quantify cell number in each reaction, qPCR was performed simultaneously for albumin gene copy number as previously described [67]. Standards were constructed for absolute quantification of gag and albumin copy number and were validated with sequential dilutions of 8E5 cell lysates that contain one copy of gag per cell. Duplicate reactions were run and template copies calculated using ABI7700 software.

### Immunohistochemistry

Ki-67 expression was assessed in formalin-fixed, paraffin-embedded lymph node samples by immunohistochemical staining as previously described [68]. Samples were incubated with monoclonal antibody against Ki-67 (Dako, Glostrup, Denmark) and goat anti-rabbit immunoglobulin G-Alexa fluor 488 (Invitrogen). The stained sections were rinsed, counterstained with hematoxylin, covered, and photographed with a Zeiss Axiophot microscope.

### Statistical Analysis

All statistical analyses were performed with Prizm (GraphPad Software, La Jolla, CA). ND and MD macaques were compared by nonparametric Mann-Whitney U test. Correlation analysis and linear regression analysis were performed between IgG^+^ percentage and log naïve/memory ratio or the naïve CD4^+^ T cell count.

## Supporting Information

Figure S1 Flow cytometric analysis of peripheral blood lymphocytes (PBMC), peripheral lymph node (PLN), and spleen samples collected at death. Naive (CD95^low^CD28^high^) and memory (CD95highCD28^high^ and CD95^high^CD28^low^) subsets of CD4^+^ T cells were analyzed by flow cytometry. Plots from six additional ND macaques (in addition to Figure 2) and two additional MD macaques demonstrate the preferential depletion of naïve CD4^+^ T cells and memory CD4^+^ T cells in ND and MD macaques, respectively.
(0.62 MB EPS)

Figure S2 Sequence alignment of the V3 loop analog of SIV in ND macaques inoculated with a molecularly cloned virus, SIVsmH635FC at 20 weeks (A) and later time points (B). Clones derived from plasma of ND macaques were compared to the CCR5 using viruses, the parental SIVsmH635FC, SIVsmE543-3, and SIVmac239 as well as the only reported CXCR4-using SIVmac/sm clone, SIVmac155T3. Amino acid substitutions are shown relative to SIVsmE543-3, conserved amino acids are indicated by a dot (.), and gaps inserted to maximize alignment are indicated by a dash (-). The virus inoculum and most of the clones have substitution in the V3 loop relative to SIVsmE543-3 at position 337, which is not associated with a switch in co-receptor usage.
(1.53 MB EPS)
